# Inflammasomes and idiopathic inflammatory myopathies

**DOI:** 10.3389/fimmu.2024.1449969

**Published:** 2024-12-11

**Authors:** Rui Sun, Jiyan Chu, Ping Li

**Affiliations:** ^1^ Department of Rheumatology, General Hospital of Northern Theater Command, Shenyang, Liaoning, China; ^2^ Graduate School, Dalian Medical University, Dalian, Liaoning, China

**Keywords:** idiopathic inflammatory myopathy, inflammasome, pyroptosis, NLR family pyrin domain containing 3 (NLRP3), absent in melanoma 2 (AIM2), caspase, gasdermin D (GSDMD)

## Abstract

Idiopathic inflammatory myopathies (IIM) are a group of systemic autoimmune diseases characterized by muscle weakness and elevated serum creatine kinase levels. Recent research has highlighted the role of the innate immune system, particularly inflammasomes, in the pathogenesis of IIM. This review focuses on the role of inflammasomes, specifically NLRP3 and AIM2, and their associated proteins in the development of IIM. We discuss the molecular mechanisms of pyroptosis, a programmed cell death pathway that triggers inflammation, and its association with IIM. The NLRP3 inflammasome, in particular, has been implicated in muscle fiber necrosis and the subsequent release of damage-associated molecular patterns (DAMPs), leading to inflammation. We also explore the potential therapeutic implications of targeting the NLRP3 inflammasome with inhibitors such as glyburide and MCC950, which have shown promise in reducing inflammation and improving muscle function in preclinical models. Additionally, we discuss the role of caspases, particularly caspase-1, in the canonical pyroptotic pathway associated with IIM. The understanding of these mechanisms offers new avenues for therapeutic intervention and a better comprehension of IIM pathophysiology.

## Background

Idiopathic inflammatory myopathies (IIMs) are a rare and diverse group of systemic autoimmune diseases, with an incidence ranging from 1 to 11 cases per million individuals (1, 2). The disease primarily presents with muscle weakness and pain in the proximal limbs, often accompanied by elevated serum creatine kinase levels. It can also affect various other organs, including the heart, lungs, skin, and digestive tract. The most common subtypes of IIM include dermatomyositis (DM), polymyositis (PM), inclusion body myositis (IBM), immune-mediated necrotizing myopathy (IMNM), and overlap myositis (3). In recent years, the identification of myositis-specific antibodies (MSAs) and myositis-associated antibodies (MAAs) has significantly improved the diagnosis and treatment of IIM (4, 5). At the same time, research suggests that abnormal activation of the innate immune system strongly influences the onset and progression of IIM (6, 7).

Pyroptosis is a lytic form of programmed cell death distinct from apoptosis and increasingly recognized for its critical role in the host’s innate immune response. Pyroptosis can occur in various cell types, including immune cells like macrophages, monocytes, and dendritic cells, as well as non-immune cells such as intestinal epithelial cells, stromal cells, hepatocytes, and myocytes (8–10). While controlled pyroptotic activity is crucial for maintaining cellular homeostasis and exerting protective functions, excessive cell death and delayed clearance of dead cells and their fragments may lead to the release of self-antigens, damage-associated molecular patterns (DAMPs), and pro-inflammatory cytokines, exaggerating immune and inflammatory responses and promoting disease development (11–14).

## Pyroptosis at glance

Our understanding of pyroptosis began in 1986 when Friedlander first observed cell death and cell content leakage in murine macrophages induced by anthrax lethal toxin (15), and this pivotal moment laid the foundation for the discovery of a new type of cell death pathway. In 1992, an enzyme initially known as ICE (interleukin-1β converting enzyme) and later identified as inflammatory caspase was found to process pro-interleukin-1β (IL-1β) into its mature form, highlighting the role of caspases in inflammation (16, 17). That same year, Zychlinsky and colleagues erroneously identified pyroptosis as apoptosis in human macrophages infected by Shigella flexneri (18). However, the true nature of this cell death process began to emerge in 1996, with reports indicating that the invasion plasmid antigen B (ipaB) of Shigella flexneri activated caspase-1, suggesting a distinct mechanism of cell death from apoptosis (19). In 2001, Cookson and Brennan first proposed the term “pyroptosis.” The term is derived from Greek, where “pyro” means fire or fever, and “ptosis” means falling. Pyroptosis is a pro-inflammatory programmed cell death characterized by pore formation, membrane rupture, and leakage of cellular contents (7, 11, 20). The introduction of this term was a crucial step in distinguishing pyroptosis from non-inflammatory apoptosis.

In 2002, the inflammasome was identified as a multiprotein complex capable of activating pro-inflammatory caspases and pro-IL-1β (21). This groundbreaking discovery linked the molecular mechanism of inflammation with cell death pathways. For a long time, pyroptosis was considered to be mediated by caspase-1. In 2011, Kayagaki and colleagues discovered that caspase-11 could induce death in mouse macrophages. This process is similar to caspase-1-mediated pyroptosis and is therefore termed “non-canonical pyroptotic pathway” (22). In 2014, Shi J and colleagues revealed that human caspase-4 and caspase-5 have the same function (23).

In 2015, gasdermin D (GSDMD) was identified as the crucial executor of pyroptosis, a milestone discovery after being cleaved by caspase-1 or caspase-4/5/11 (24). This, along with the subsequent definition of pyroptosis as gasdermin-mediated programmed cell death (25, 26), has fundamentally changed our understanding of cell death mechanisms (**Table 1**). In 2017, Shao’s research group found that chemotherapeutic drugs can induce pyroptosis by triggering caspase-3-mediated cleavage of GSDME (29). The following year, it was discovered that caspase-8 also cleaves GSDMD, thereby initiating pyroptosis during Yersinia infection (30, 31). In 2020, it was found that pyroptosis can also be induced independently of caspase, significantly changing our understanding of this process. Granzyme A (GZMA) secreted by cytotoxic T lymphocytes and natural killer (NK) cells cross-domain cleaves GSDMB specifically, activating GSDMB-dependent pyroptosis in malignant cells (32). Similarly, granzyme B (GZMB) from these immune cells can also initiate GSDME-dependent apoptotic cell death in tumor cells through caspase-3-mediated cleavage of GSDME (33).

**Figure 1:**
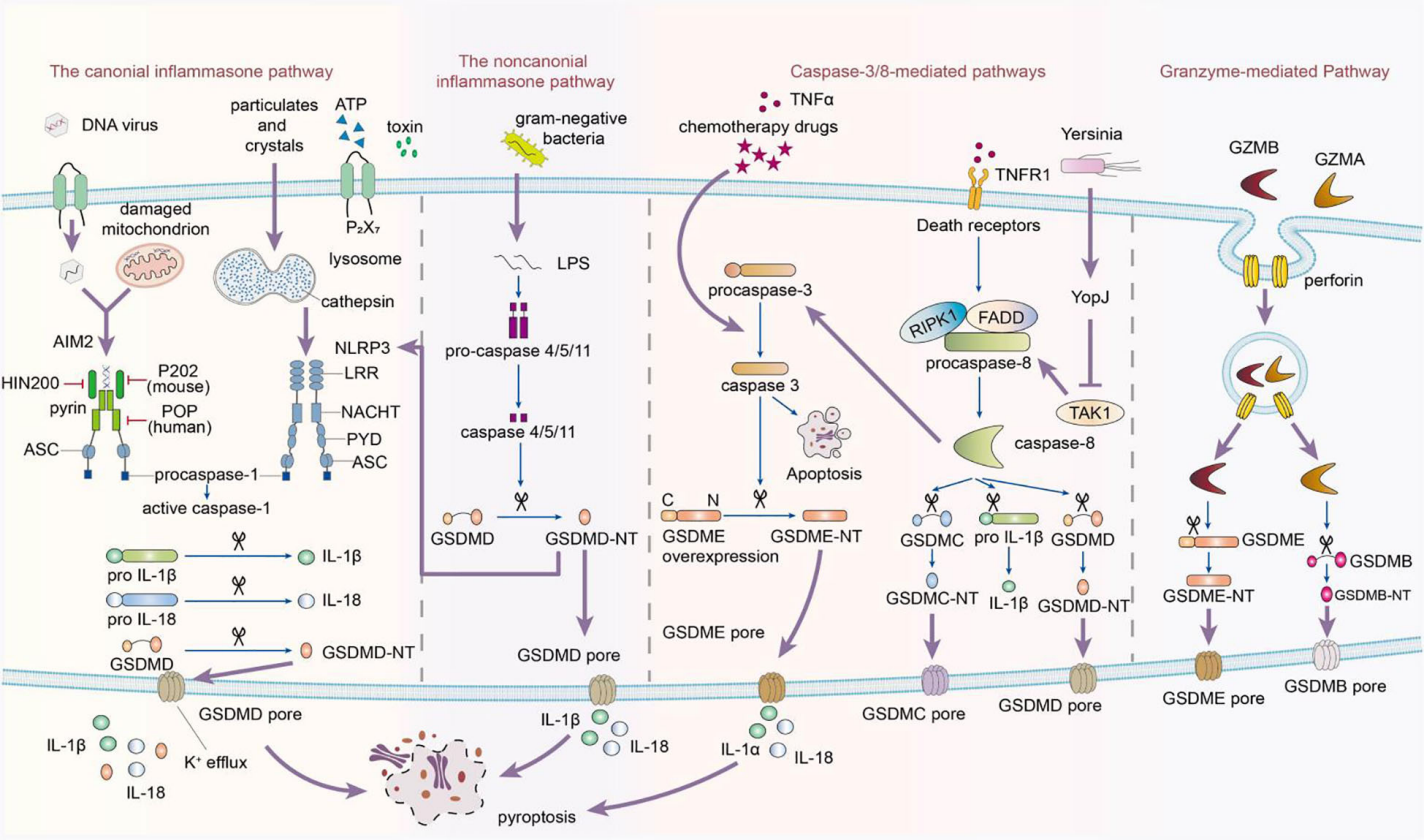
This image depicts the activation of the inflammasome, which follows a biphasic signaling pattern. The process begins with a priming signal initiated by pattern recognition receptors. There are four recognized pathways in signal activation. The canonical pathway depends on Caspase-1, while the noncanonical signaling cascade relies on Caspase-4/5/11. Caspase-1, -4, -5, and -11 selectively cleave GSDMD, releasing its N-terminal fragments to form transmembrane pores in the cytoplasmic lipid bilayer, leading to pyroptosis. Additionally, Caspase-3 can induce pyroptosis in incomplete pathways by cleaving GSDME when cells highly express GSDME in response to tumor necrosis factor-alpha (TNF-α). Conversely, in cells with negative or low GSDME expression, Caspase-3 triggers apoptosis. In the caspase-8-mediated pathway, inhibiting TAK1 induces the activation of caspase-8, which cleaves GSDMD, resulting in pyroptosis. In the granzyme-mediated pathway, CAR T cells rapidly activate caspase-3 in target cells by releasing GZMB, which then activates GSDME, causing extensive pyroptosis. Additionally, GZMA and GZMB in cytotoxic lymphocytes enter target cells through perforin and induce pyroptosis. GZMA hydrolyzes GSDMB, and GZMB directly activates GSDME.

The journey through the history of pyroptosis has been marked by significant discoveries that have deepened our understanding of cell death and its role in immune responses (**Figure 1**). As research continues, the intricate mechanisms and regulatory pathways of pyroptosis will likely reveal further insights into the complex interplay between cell death and inflammation. In particular, recent studies have identified the contribution of inflammasomes to the pathogenesis of immune-related myopathies (IIM), and this article aims to summarize the research findings on this topic.

## Inflammasomes

The inflammasome is a complex composed of multiple proteins, formed in response to immune signals from pathogen-associated molecular patterns (PAMPs) and damage-associated molecular patterns (DAMPs), playing a key role in coordinating host immune responses (27). The inflammasome sensor complex is composed of two receptor families: the nucleotide-binding oligomerization domain-like receptors (NLRs), including NLRP1,NLRP3,NLRP6,NLRP7,NLRC4, NLRP10 and NLRP12, and the PYHIN family containing the HIN domain, including AIM2 and pyrin (28) (**Table 1**).

**Table 1:** Classification, domains, and common activators of inflammasome component protein (27, 28).

Classification	Sensor protein	Effector domain	Common activators
NLRs family	NLRP1	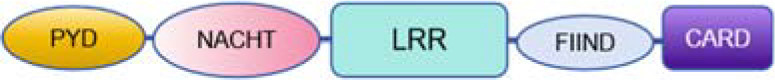	anthracis lethal toxin, DPP8/9 inhibitors tegument protein ORF45
	NLRP3	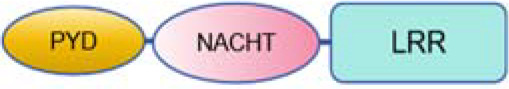	ATP, ion flux ( K+ efflux), particulate matter, ROS, pathogen-associated RNA, bacterial and fungal toxins, and component
	NLRC4	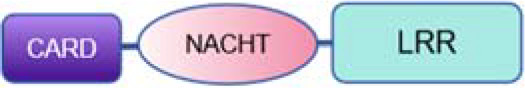	Gram-negative bacteria and flagellin, the type III or IV secretion system proteins
	NLRP6	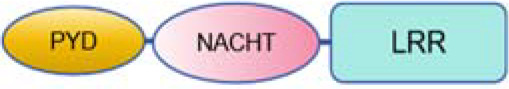	Gram-positive, bacteria-derived lipoteichoic acid、LPS、viral infections
	NLRP7	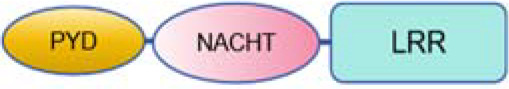	mycoplasma and Gram-positive bacterial infections
	NLRP10	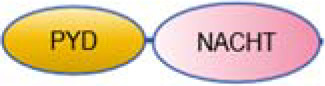	phospholipase C activator 3m3-FBS
	NLRP12	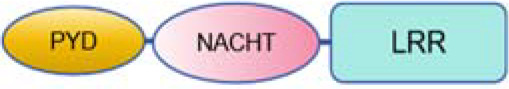	Yersinia pestis infection
PYHIN family	AIM2	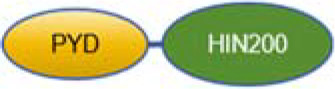	cytosolic dsDNA of viral, bacterial, or host origin
	Pyrin	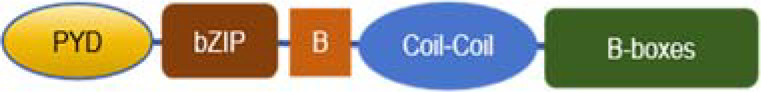	RhoA inhibiting microbial toxins
ASC protein	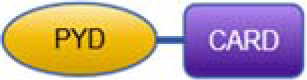		
Pro-caspase	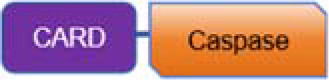		

Currently, most research on inflammasomes converged on infection and cancer fields. Due to the rare nature of the disease, research on idiopathic inflammatory myopathies(IIMs) is relatively limited, mainly converged on NLRP3 and AIM2. Exploring pyroptosis mechanisms in IIMs remains an underdeveloped area, with many knowledge gaps needing to be filled. Here, we review the latest progress of inflammasomes in patients with IIMs.

## The role of NLRP3 inflammasome in IIMs

NLRP3 inflammasome is a multiprotein complex that plays a crucial role in the innate immune response. Due to its involvement in various inflammatory diseases, including idiopathic inflammatory myopathies (IIM), this inflammasome has been extensively studied (34). NLRP3 is expressed in monocytes, neutrophils, dendritic cells, lymphocytes, epithelial cells, and muscle cells. It consists of the NLRP3 protein, the adaptor protein ASC, and pro-caspase-1. The NLRP3 protein is a 115 kDa cytosolic protein, containing three domains: the C-terminal leucine-rich repeat (LRR) domain that senses danger signals, the nucleotide-binding and oligomerization domain (NACHT) with ATPase activity, and the N-terminal PYD domain that interacts with ASC (34). The structure of the NLRP3 inflammasome has been described as ring-shaped, with a curved LRR and a globular NACHT domain. Under normal physiological conditions, the interaction between the NACHT and LRR domains keeps NLRP3 in a self-inhibited state. However, cellular stress factors, whether pathogen-associated molecular patterns (PAMPs) or damage-associated molecular patterns (DAMPs), can disrupt this balance (34, 35). Upon activation, NLRP3 undergoes conformational changes, leading to self-cleavage to form P20 and P10 subunits. The NACHT domain plays a critical role in ATP binding and hydrolysis. The P20 subunit contains the LRR domain, while the P10 subunit contains the NACHT and PYD domains. This cleavage and subsequent interaction between the PYD domain of NLRP3 and ASC are key steps in assembling the inflammasome complex, resulting in the recruitment and activation of pro-caspase-1. Once activated, caspase-1 processes the pro-forms of IL-1β and IL-18, leading to their maturation and secretion, thereby propagating the inflammatory response.

NLRP3 inflammasome is associated with various diseases, including metabolic syndrome such as obesity, type 2 diabetes (T2D) (36), and gout (37), neurodegenerative diseases such as Alzheimer’s disease (AD) (38) and multiple sclerosis (MS) (39), inflammatory bowel diseases (IBD) such as Crohn’s disease and ulcerative colitis (40, 41), cardiovascular diseases (42), autoimmune conditions like systemic lupus erythematosus (SLE) (43–45) and rheumatoid arthritis (RA) (46), various cancers (47), and infections including viral, bacterial, and fungal pathogens (11). The role of NLRP3 inflammasome in these diseases highlights its importance in immune responses and inflammation. NLRP3 inflammasome is also involved in the pathogenesis of idiopathic inflammatory myopathy through multiple mechanisms.

## Canonical pathway

Canonical activation involves two steps. The initial priming step is promoted by pattern recognition receptors such as Toll-like receptors and cytokine receptors (e.g., IL-1R, TNFR) on the membrane, triggering NF-κB nuclear translocation, thereby enhancing the expression of NLRP3 and pro-IL-1β at the transcription and translation levels (48). Recently, many studies have provided strong evidence indicating that the priming step is not limited to transcriptional upregulation, post-translational modifications (PTMs) of NLRP3 protein, such as ubiquitination and phosphorylation, also play a critical role in the activation of the NLRP3 inflammasome (49–51). The next step is the activation step, where secondary signals can be induced by various stimuli, including microbial invasion, exotoxins, extracellular ATP, crystals, and double-stranded DNA, leading to oligomerization of the NLRP3 inflammasome and activation of caspase-1 (52–63). Once activated, caspase-1 is responsible for the proteolytic cleavage of pro-inflammatory cytokines IL-1β and IL-18, converting them into their mature biologically active forms. Increasing evidence emphasizes the importance of NLRP3 inflammasome activation, caspase-1 activation, and IL-1β release in the development of autoimmune diseases such as systemic lupus erythematosus, multiple sclerosis, and ulcerative colitis (39–41, 43–45).

Recent research has revealed the significance of NLRP3 inflammasome signaling cascade in idiopathic inflammatory myopathies. Yin Xi’s research group reported that compared to healthy controls, patients with polymyositis (PM) and dermatomyositis (DM) showed elevated levels of NLRP3, IL-1β, and IL-18 mRNA in muscle fiber tissues, as well as increased expression of NLRP3 and caspase-1 p20 protein subunit (64). The study indicates that in DM/PM patients, the high expression of NLRP3 inflammasomes in muscle tissues activates caspase-1 expression, leading to upregulation of IL-1β and IL-18 levels, enhancing cellular immunity. There was no significant difference in the expression levels of NLRP3 and caspase-1 between DM and PM patients, possibly due to NLRP3 inflammasomes being involved in local muscle inflammation in DM/PM, with the released IL-1β/IL-18 possibly exacerbating disease progression (64). The activation of NLRP3 inflammasomes in DM/PM is attributed to muscle fiber necrosis, releasing high mobility group box-1, ATP, and hyaluronic acid as damage-associated molecular patterns, which may serve as activators of NLRP3 inflammasomes (63, 65). Additionally, Xia P and colleagues assessed the role of NLRP3 inflammasomes in PM using an experimental rat model and C2C12 myoblast cells (66). They observed a significant upregulation of NLRP3, caspase-1, and IL-1β in affected muscles, and treatment with NLRP3 inflammasome inhibitor MCC950 or NLRP3-specific siRNA reduced inflammation and improved muscle function, highlighting the importance of the NLRP3/caspase-1/IL-1β Canonicalal pathway in PM.

## Non-canonical pathway

The non-Canonical activation of NLRP3 inflammasome occurs in one step, bypassing the induction phase. In a mouse model of experimental autoimmune myositis (EAM), researchers observed elevated levels of NLRP3, caspase-4, caspase-5, caspase-11, pannexin-1, ATP-activated P2X7 receptor (P2X7R), and GSDMD mRNA and protein in muscle tissue, accompanied by increased serum concentrations of IL-1β and IL-18 (67). Lipopolysaccharide (LPS) from gram-negative bacteria activates caspase-4/5/11, promoting the release of ATP through the pannexin-1 channel. This ATP subsequently activates the P2X7R channel, leading to potassium (K+) efflux. These signals trigger NLRP3 inflammasome, resulting in the secretion of IL-1β and IL-18 and the induction of pyroptosis (23). This process disrupts the ion balance of the cell membrane, leading to membrane rupture, release of intracellular inflammatory contents, and initiation of an inflammatory response. ATP and K+ also act as secondary messengers to activate NLRP3 inflammasome, enhancing the production of inflammatory factors, and potentially exacerbating pyroptosis, inflammation, and muscle damage.

Glyburide is a sulfonylurea antidiabetic drug known to block ATP-sensitive potassium channels on pancreatic β cells (68). Previous studies have shown that in conditions such as renal impairment (69), sepsis-induced myocardial injury (70), and asthma (71), glyburide reduces inflammation by inhibiting NLRP3 activation and decreasing levels of inflammatory mediators such as IL-1β and IL-18 (72). Following glyburide intervention, expression of NLRP3, IL-1β, and IL-18 in skeletal muscle tissues of experimental autoimmune myocarditis (EAM) mice is reduced, muscle strength is improved, and scabs form at sites of muscle rupture and abscess (67). These results suggest that the NLRP3 inflammasome-mediated IL-1β/IL-18 pathway plays a role in the pathogenesis of idiopathic inflammatory myopathy, and glyburide inhibiting this pathway leads to reduced secretion of inflammatory factors, alleviation of inflammation, and improvement in muscle strength. Glyburide may become a novel targeted therapeutic drug for controlling or treating IIMs (67).

Bright blue G (BBG), also referred to as Coomassie blue, is a potent antagonist of P2X7R (67). Studies have shown that intravenous injection of BBG can improve recovery in mice with spinal cord injury and reduce local inflammation (73). BBG can also significantly reduce cardiac fibrosis by lowering the expression levels of NLRP3, IL-1β, and caspase-1 (74). Following BBG intervention, the levels of P2X7R, NLRP3, and IL-1β proteins, as well as P2X7R mRNA in the muscles of EAM mice, decreased, while IL-18 levels remained unchanged. In addition, new hair growth appeared in the alopecia area compared to before the intervention, and muscle strength increased. Previously ruptured and suppurative ulcers began to scab over, and new granulation tissue appeared (67). This indicates that P2X7R may play a role in the development of IIM through the NLRP3 inflammasome/IL-1β pathway. BBG inhibits the P2X7R-mediated NLRP3/IL-1β pathway, reduces inflammation, and gradually restores function.

In summary, glyburide and BBG are promising therapeutic agents for the treatment of IIMs. However, these findings are based solely on experiments in animals, further research is needed to confirm their efficacy and safety in clinical settings.

## Interactions of NLRP3 inflammasome with other pathways

### Metabolic pathway

The rapidly developing field of immunometabolism delves deep into the intricate interplay between metabolic reprogramming and immune system function, providing new insights into immune responses in health and disease (75). Cellular metabolism involves six major pathways: glycolysis, tricarboxylic acid (TCA) cycle, fatty acid oxidation, fatty acid synthesis, amino acid metabolism, and pentose phosphate pathway (76). Glycolysis converts glucose to pyruvate and generates adenosine triphosphate (ATP). Under normal oxygen conditions, pyruvate enters the TCA cycle and undergoes oxidative phosphorylation, while under low oxygen conditions, it is converted to lactate (77).

Skeletal muscle is a key component in glucose uptake, containing numerous energy-sensing molecules that respond to changes in energy homeostasis in pathological conditions (78). Intrinsic immune receptors on muscle cells and immune cells, such as Toll-like receptors and nucleotide-binding oligomerization domain-like receptors, can influence muscle metabolism through intercellular communication (79–81). Mass spectrometry and bioinformatics studies have shown disturbances in various glycolytic processes, NLRP3 inflammasomes, and programmed cell death pathways in muscle tissues of patients with DM/PM (82). Upregulation of pyruvate kinase M2 (PKM2) was observed in muscle fibers of DM/PM patients and was positively correlated with the expression levels of NLRP3 inflammasomes (82).

In addition, a protein related to pyroptosis is elevated in DM/PM muscle tissues, with a more pronounced effect in PM. In myositis patients expressing anti-signal recognition particle autoantibodies, levels of PKM2 in muscle tissue and interleukin-1β (IL-1β) in plasma are high, indicating a link between metabolic imbalance and immune activation. The PKM2 inhibitor shikonin has been found to reduce the activation of NLRP3 inflammasomes in muscle fibers stimulated by IFNγ, reducing pyroptotic cell death, underscoring the role of NLRP3 inflammasomes in the pathogenesis of IIM (82).

### Neutrophil extracellular traps pathway

In recent years, research has revealed that neutrophil dysregulation is a driving factor behind IIMs (83, 84). NETs serve as biomarkers for disease activity and are crucial in the pathogenesis of IIMs (85, 86). When stimulated, neutrophils form a DNA-based protein network, including histones, myeloperoxidase (MPO), neutrophil elastase, antimicrobial peptide LL-37, matrix metalloproteinase 9 (MMP9), and other granular proteins, which eliminate pathogens (87, 88). This process is known as NETosis (89–92). NETs undeniably enhance host defenses by capturing pathogens. However, excessive activation and insufficient degradation of NETs can worsen inflammation, and tissue damage, and cause autoimmune diseases like IIMs (86, 93, 94). NETosis releases nuclear contents into the extracellular space, akin to damage-associated molecular patterns (DAMPs). In autoimmune diseases such as IIM, excessive NET formation leads to tissue damage and disease progression. In IIM-ILD, NETs can harm pulmonary vascular endothelial cells, increasing vascular permeability and causing edema (95). By promoting lung fibroblast proliferation and differentiation into myofibroblasts, NETs contribute to pulmonary fibrosis via the TLR9-miR-7-Smad2 pathway (96). NETs can activate the NLRP3 inflammasome, resulting in the release of IL-1β and other pro-inflammatory cytokines, amplifying the inflammatory response in IIM (97). Experimental autoimmune myositis (EAM) mouse models have provided insights into NETs’ role in IIM-ILD (98). These models show increased NET formation following immunization with skeletal muscle homogenate and pertussis toxin (PTX) (98), with NET presence in the lungs correlating with ILD severity, indicating a pathogenic role for NETs.

Given NETs’ role in IIM-ILD, targeting their formation or degradation could offer new therapeutic strategies. Small molecules or biologics that inhibit NET release by neutrophils might reduce tissue damage and inflammation in IIM (99, 100). Enhancing DNase I activity or other enzymes degrading NETs could mitigate excessive NET formation effects (101). Targeting the NLRP3 inflammasome or other downstream pathways activated by NETs might provide additional therapeutic benefits (102–104). Further research is required to fully understand NETs’ contribution to IIM pathogenesis and develop targeted therapies for managing IIM-ILD. Future studies should investigate specific NET components, genetic factors influencing NET formation, and novel therapeutic agents that modulate NET-associated pathways.

### Autophagy and endoplasmic reticulum stress pathways

NLRP3 inflammasome can interact with other cellular pathways, such as autophagy and endoplasmic reticulum (ER) stress, which can be particularly relevant in muscle cells under metabolic stress (105–107). The dysregulation of these pathways can lead to the accumulation of damaged organelles and protein aggregates, which can activate the NLRP3 inflammasome (107).

Inclusion body myositis (IBM) is a complex muscle disease characterized by progressive muscle weakness and atrophy, significantly impacting the quality of life of affected individuals. The pathogenesis of IBM is not fully understood, but it has been recognized that both inflammatory and degenerative mechanisms play a role in the onset and progression of the disease. In IBM, NLRP3 inflammasome is upregulated, and its activation is associated with the accumulation of characteristic protein aggregates (107). These aggregates include β-amyloid proteins, tau proteins, and others found in neurodegenerative diseases, suggesting a potential common pathological mechanism (108).

Autophagy is the cellular process responsible for degrading and recycling damaged proteins and organelles. Dysfunction of autophagy is associated with IBM as it may lead to the accumulation of protein aggregates and promote muscle fiber degeneration. The NLRP3 inflammasome can be activated by dysfunctional autophagy activity and reactive oxygen species (ROS) that play a significant role in IBM pathophysiology. Therefore, there is a reciprocal relationship between autophagy and activation of the NLRP3 inflammasome, and they can mutually influence each other (109).

Endoplasmic reticulum(ER) stress is another key factor in the pathogenesis of IBM. The ER is responsible for protein folding and modifications. When its function is impaired, it may lead to the accumulation of misfolded proteins, which can aggregate. NLRP3 inflammasome can be activated by ER stress. Conversely, activation of NLRP3 inflammasome may further impair ER function, forming a vicious cycle, and exacerbating muscle fiber damage and inflammation (108).

The upregulation of NLRP3 in IBM is associated with inflammatory cytokines and ubiquitin (a marker of protein degradation) levels (109). This indicates that the complex interactions between NLRP3 inflammasome, autophagy, and ER stress may be key driving factors in the pathogenesis of IBM. However, further studies are needed to elucidate the exact roles of these pathways in the pathogenesis of IBM. Understanding the complex interactions between NLRP3 inflammasome, autophagy, and ER stress may provide new insights for therapies for IBM and other muscle diseases characterized by protein aggregation and inflammation.

## NLRP3 inhibitors

In DM, PM, IBM, and anti-synthetase syndrome, the NLRP3 inflammasome has been shown to play a central role. Targeting the NLRP3 inflammasome may bring new hope for the treatment of IIM. Several types of NLRP3 inhibitors have been identified, including Glyburide, synthetic small molecules such as MCC950, JC124, BHB, CY-09, and OLT1177, natural products such as Shikonin, Parthenolide, Tranilast, and Oridonin (**Table 2**). These inhibitors have shown high efficiency and specificity in inhibiting NLRP3 activation, providing valuable tools for studying the pathogenesis of diseases (123–125). Pharmacological modulation of NLRP3 inflammasomes has been explored in preclinical models of diseases such as atherosclerosis (126), gout (127), and inflammatory bowel disease (128), providing promising evidence of the potential of NLRP3 inhibitors in the treatment of inflammatory diseases.

**Table 2:** Structure, target, and mechanism of potential inhibitors of NLRP3 inflammasome.

Agent	Structure	Target	Potential mechanism	References
Gyburide	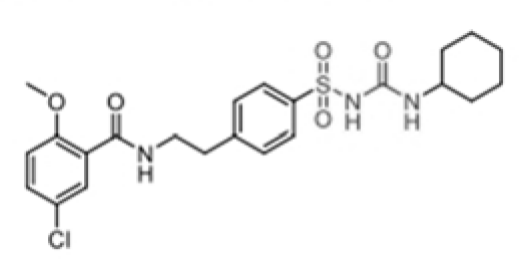	NLRP3 (indirectly)	Inhibits ATP-sensitive K+channels; Downstream of P2X7 resulting in inhibition of ASO aggregation.	(110)
MCC950	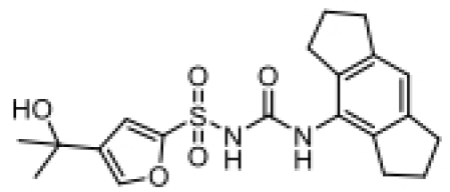	NLRP3	Alkylates the cysteines in the ATPase domain of NLRP3,inhibitsNLRP3 ATPase activity.	(111, 112)
JC124	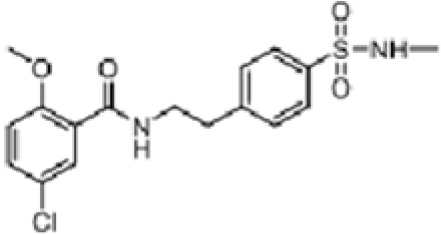	NLRP3?	Blocks the expression of NLRP3, ASC, caspase-1, pro-1L-1β, TNFα and iNOS.	(113, 114)
β-hydroxybutyrate (BHB)	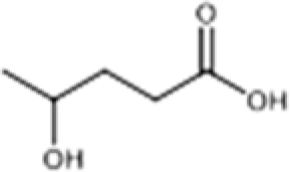	NLRP3 (indirecty)	Covalent modification of the catalytic cysteine residue in the active site caspase-1 resulting in caspase-1 blocking and resulting of pro-IL-1β/18.	(115)
CY-09	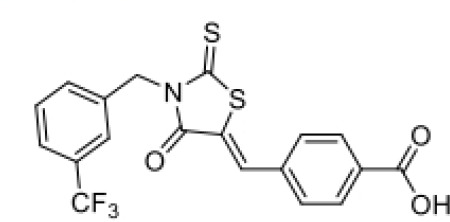	NLRP3	Blocks the ATPase domain of NLRP3 resulting in inhibition of canonical and non-canonical NLRP3 inflammasome activation.	(116)
OLT1177	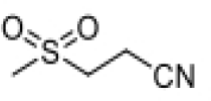	NLRP3	Inhibits NLRP3 ATPase activity, and blocks NLRP3 inflammasome activation.	(117)
Shikonin	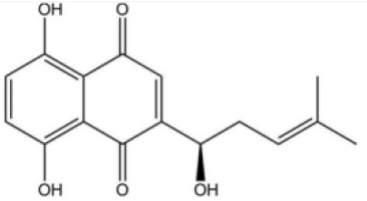	NLRP3 (indirectly)	inhibiting the activation of PKM2	(118)
Parthenolide	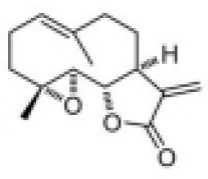	NLRP1,NLRP3	Suppresses IL-1β/18 release.	(119, 120)
Tranilast	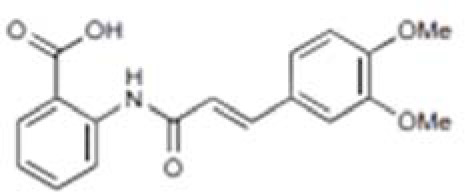	NLRP3 (indirecty)	Inhibits NLRP3 ATPase activity by cysteine modification, blocks NLRP3inflammasome activation.	(121)
Oridonin	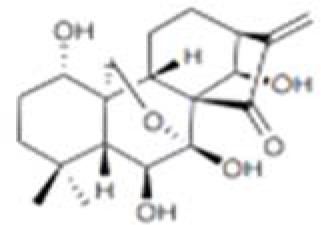	NLRP3	Inhibits NLRP3 ATPase activity, blocks NLRP3 inflammasome activation. Binds to cysteine of NLRP3 to abolish NLRP3-NEK7 interaction, blocks NLRP3 inflammasome activation.	(122)

Glyburide is a sulfonylurea antidiabetic drug that can block the ATP-sensitive K+ channels on pancreatic β-cells (129). Recent studies have reported its potential anti-inflammatory effects, which can inhibit the expression of NLRP3 inflammasome (129). Following glyburide intervention, the expression levels of NLRP3, IL-1β, and IL-18 in mice decreased, muscle strength improved, and ulcers and purulent sites began to scab (68). These findings suggest that the NLRP3 inflammasome-mediated IL-1β/IL-18 pathway is related to the pathogenesis of IIMs. The hypoglycemic side effects of glyburide limit its widespread use, therefore, the development of glyburide analogs is expected to become a new targeted drug for controlling or treating IIM.

MCC950 is a sulfonylurea derivative and a selective inhibitor of NLRP3, specifically inhibiting the activation of NLRP3 without affecting AIM2, NLRC4, or NLRP1 inflammasomes, with no risk of hypoglycemia (123, 130). Previous reports have indicated that MCC950 can block the processing of IL-1β by caspase-1 (123, 130), and subsequent studies have suggested its potential to simultaneously inhibit both canonical and non-canonical NLRP3 inflammasome activation and IL-1β production by blocking ASC aggregation (123, 130). In the context of IIM, MCC950 has been shown to reduce the overexpression of MHC-I in C2C12 cells co-cultured under LPS/ATP challenge (67). *In vitro* experiments, pretreatment with MCC950 effectively inhibited NLRP3 inflammasome and pyroptosis activation in human pulmonary microvascular endothelial cells (HPMECs) stimulated by NETs (110). *In vivo*, inhibition of NLRP3 inflammasome by MCC950 reduced the expression of NLRP3, IL-1β, and MHC-I in muscle tissues of PM model rats, alleviating the severity of muscle inflammation and the levels of CRP, CK, and LDH in serum (65). MCC950 effectively inhibited the activation of NLRP3 inflammasomes in lung microvascular endothelial cells (PMECs) in a mouse model of experimental autoimmune myositis (110). The anti-inflammatory and anti-pyroptotic effects of MCC950 have also been confirmed in Duchenne muscular dystrophy muscle specimens.

Shikonin is a traditional Chinese medicine isolated from Lithospermum erythrorhizon, a plant of the Lithospermum genus, and has anti-cancer and anti-inflammatory properties (111). As a PKM2 inhibitor, Shikonin can prevent muscle cell pyroptosis by indirectly inhibiting the activation of the NLRP3 inflammasome (82, 111), potentially providing therapeutic benefits in idiopathic inflammatory myopathies.

Some other NLRP3 inhibitors have been studied for other metabolic and autoimmune diseases, but have not been tested in the context of IIM. JC124 is an innovative sulfonamide analogue that has been shown to provide significant anti-inflammatory benefits in the context of traumatic brain injury (112), acute myocardial infarction, and Alzheimer’s disease (131). The compound demonstrates its efficacy by significantly downregulating the expression of key inflammatory mediators [including NLRP3, ASC, caspase-1, pro-IL-1β, TNFα, and inducible nitric oxide synthase (iNOS)], all of which are critical steps in the inflammatory cascade. Beta-hydroxybutyrate (BHB) is a ketone body that supports mammalian cell metabolism during energy deficiency, serving as an alternative source of ATP and exerting its antioxidant effects, with neuroprotective properties (118). In 2015, Youm et al. reported that BHB can inhibit the activation of the NLRP3 inflammasome without affecting the activation of NLRC4, AIM2, or the non-Canonicalal caspase-11 inflammasome, and inhibits NLRP3 inflammasome by preventing K+ efflux, reducing ASC aggregation, and speck formation (118). CY-09 is a small molecule compound specifically developed to inhibit the assembly and activation of the NLRP3 inflammasome by binding to the ATP binding site of the NLRP3 NACHT domain, suppressing NLRP3 ATPase activity (113). It has shown excellent therapeutic effects *in vivo* in CAPS and T2D mouse models. OLT1177 is a β-sulfonyl nitrile identified as a drug candidate for the treatment of osteoarthritis and acute gouty arthritis (114). It inhibits the activity of NLRP3 ATPase, blocking the activation of the NLRP3 inflammasome, with no effect on the NLRC4 and AIM2 inflammasomes (114). It has potential in the treatment of NLRP3-related diseases. Parthenolide is a sesquiterpene lactone derived from the plant Tanacetum parthenium. It reduces NLRP3 ATPase and caspase-1 activity by inhibiting NF-kB (115, 116). Tranilast, a tryptophan metabolite analogue, inhibits NLRP3 ATPase activity through cysteine modification and blocks the activation of NLRP3 inflammasome (117). Oridonin, a bioactive diterpenoid, mainly derived from the herbal plant Rabdosia rubescens, eliminates the interaction between NLRP3 and NEK7 by binding to the cysteine of NLRP3, thus blocking the activation of NLRP3 inflammasome (119).

It is looking forward to more research to elucidate the therapeutic potential of these inhibitors in IIM.

## AIM2 inflammasome in IIM

The absence of melanoma 2 (AIM2), a member of the AIM-like receptor (ALR) family, plays a critical role in the intracellular DNA innate immune response. It is a 39 kDa protein composed of 344 amino acids, and the encoding gene AIM2 is located in the q22 region of human chromosome 1 (120, 121). Cloned in 1997 as a tumor suppressor, AIM2 gained attention only in 2009. It is characterized by an N-terminal pyrin domain (PYD) and a C-terminal HIN-200 domain, also known as the PYHIN domain, which is crucial for recognizing double-stranded DNA (dsDNA) (122, 132). Upon dsDNA recognition, AIM2 initiates the assembly of ASC, leading to the proteolytic activation of pro-caspase-1. This activation is essential for the AIM2 inflammasome complex, with significant implications for regulating inflammatory responses (133–136). AIM2’s sensitivity to dsDNA is highly dependent on the length and sequence of DNA, with the optimal response occurring between 80 and 200 base pairs (137).

In addition to tissue damage caused by various infections, radiation, systemic lupus erythematosus, and rheumatoid arthritis (138–140), AIM2 also plays an important role in the progression of myositis (141). Loell et al. demonstrated through transcriptome microarray technology and immunoblotting that immunosuppressive therapy can downregulate the expression of AIM2 and caspase-1 in skeletal muscle samples from patients with DM and PM, suggesting that this may be a potential therapeutic strategy to reduce inflammation in IIM patients. The balance between the anti-inflammatory effects and the negative impact on muscle remodeling must be considered (141).

## Interactions of AIM2 inflammasome with other pathways

### cGAS-STING pathway

The activity of AIM2 is regulated by its interaction with other innate immune sensors and cellular homeostasis. It interacts with the cGAS-STING pathway, which triggers type I interferon responses in response to cytoplasmic dsDNA. While the cGAS-STING signaling pathway can promote the initial activation of AIM2 inflammasomes, the AIM2 inflammasomes can also inhibit the cGAS-STING signaling pathway through the disruption of intracellular potassium levels caused by caspase-1-mediated cleavage of cGAS and GSDMD pore formation (142). Baatarjav et al. found that AIM2 deficiency leads to macrophage accumulation and impaired renal function recovery, and further studies revealed that AIM2 deficiency exacerbates inflammation through the STING-TBK1-IRF3/NF-κB signaling pathway, even in the absence of IL-1β mobilization (143).

### AIM2 interaction with NLRP3

Multiple reports indicate that these two inflammasomes are essential in many inflammatory responses, including bacterial infections, malaria parasite infections, and STING agonist stimulation (144–146). AIM2 is also involved in a form of mixed cell death pathway called PANoptosis, which simultaneously activates pyroptosis, programmed cell death, and necrotic cell death, where AIM2 has been reported to form a complex with pyrin and ZBP1 to execute this form of inflammatory cell death (147). Furthermore, changes in cellular metabolism also affect AIM2 responses. For example, excess cholesterol can trigger the release of mitochondrial DNA (mtDNA) and the activation of AIM2 (148). There are reports indicating that upregulation of the glycolytic enzyme pyruvate kinase M2 subtype in macrophages initiates the response of AIM2 and NLRP3 inflammasomes (149).

## AIM2 inhibitors

In the process of searching for AIM2 inflammasome inhibitors, various natural products have shown potential (**Table 3**). The diterpene lactone andrographolide from Andrographis paniculata has been suggested to inhibit the activation of the AIM2 inflammasome by preventing AIM2 translocation to the nucleus (158), significantly improving radiation-induced lung injury and alleviating the progression of radiation pneumonitis and lung fibrosis (141). Extracts from Cornus officinalis and Panax ginseng, known as ginsenosides, have been shown to inhibit the secretion of IL-1β and IL-18 induced by AIM2 inflammasome activation, as well as the aggregation of ASC and cleavage of Gasdermin D (159, 160). EFLA945 is a water-soluble extract from Vitis amurensis leaves containing resveratrol and paeoniflorin 3-O-glucoside, which may inhibit AIM2-dependent IL-1β secretion associated with the pathogenesis of psoriasis (150). Quercetin, a flavonoid with anti-inflammatory properties, has been shown to downregulate the expression of AIM2 and pro-caspase-1 in keratinocytes stimulated by IFN-γ and poly(dA:dT), thereby inhibiting the JAK2/STAT1 pathway and reducing inflammation caused by inflammatory skin diseases (155, 156).

**Table 3:** Structure, target, and mechanism of potential inhibitors of AIM2 inflammasome.

Agent	Structure	Target(s)	Potential mechanism	References
andrographolide	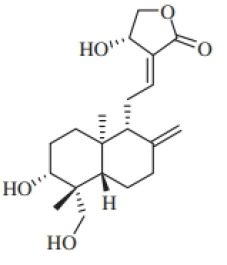	AIM2 inflammasome activation	preventing AIM2 translocation to the nucleus	(144, 150)
EFLA 945 (resveratrol )	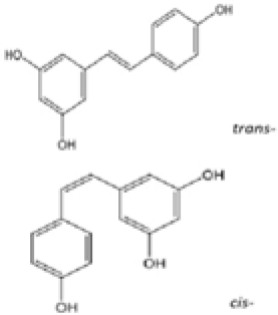	AIM2 inflammasome	restrict the AIM2 inflammasome activation through preventing DNA entry	(151)
Quercetin	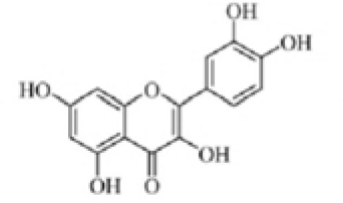	NLRP3 inflammasomes, AIM2 inflammasomes direct inhibitor	Phosphorylate ASC and inhibit pro- caspase-1 recruitment	(152, 153)
RGFP966	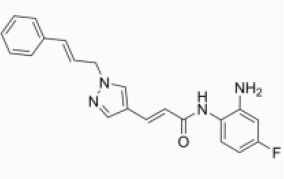	AIM2 inflammasomes	selectively inhibit histone deacetylase 3, modulates the acetylation and phosphorylation of STAT1 to suppress AIM2,	(154)
Cornus officinalis Seed Extract	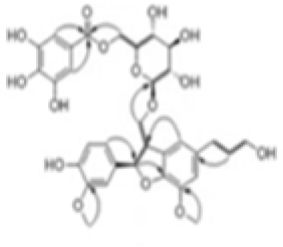	AIM2 inflammasomes	inhibited the cleavage of caspase-1, the translocation and speck formation of ASC,inhibit AIM2 speck formation	(155)
Ginsenoside	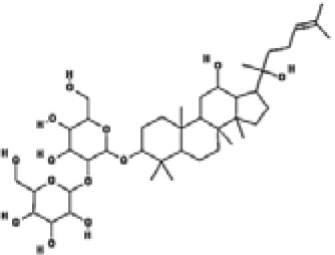	NLRP3 inflammasome AIM2 inflammasome (predominant)	Attenuate IL-1β secretion as well as pathogen clearance ,inhibit AIM2 inflammasome activation.	(156)
4-sulfonic Calixarenes	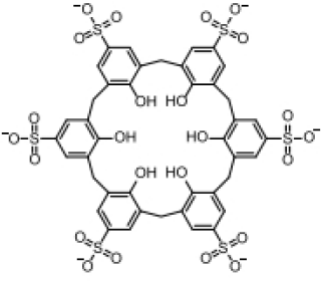	cGAS,TLR9, AIM2 inflammasomes (direct inhibitor)	Bind completelyto the DNA binding inflammasomes site through exposed sulfonic acid group to suppress AIM2 inflammasomes formation	(157)

A group of compounds also showed inhibitory effects on the AIM2 inflammasome, demonstrating their therapeutic potential (**Table 3**). RGFP966 is a selective inhibitor of histone deacetylase 3 that inhibits AIM2 by regulating the acetylation and phosphorylation of STAT1, thereby alleviating ischemic brain injury (151). 4-sulfonated macrocyclic compounds bind to the DNA-binding inflammasome site, inhibiting the formation of the AIM2 inflammasome through exposed sulfonate groups (152). Synthetic oligonucleotides, super ODNs containing the TTAGGG sequence, can compete with dsDNA to bind to the HIN200 domain of AIM2, thereby inhibiting the activation of the AIM2 inflammasome (153). POP3 can directly bind to the PYD domain of AIM2, prevent interaction with ASC, and inhibit the secretion of IL-1β (154).

Inhibitors targeting AIM2 have shown great potential in treating autoimmune diseases. Future research is expected to explore the role of AIM2 in the pathogenesis of IIMs, and the mechanism of action, efficacy, and safety of AIM2 inhibitors.

## Caspases

Caspases are a family of endopeptidases that specifically cleave their substrates at aspartic or glutamic acid residues, playing a pivotal role in the regulation of cellular processes (157). In mammalian cells, this family encompasses at least 14 distinct members (161–163). They are broadly classified into two functional groups: apoptotic caspases and inflammatory caspases, as detailed in **Table 4**.

**Table 4:** Classification, Structure and Function of Caspases (164–166).

Classification		Caspase	Structure	Function
inflammatory caspase	initiator caspase	caspase-1	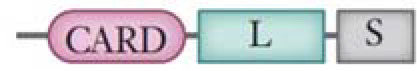	It can cleave a variety of substrates, including pro-IL-1β, pro-IL-18, and GSDMD.
		caspase-4h/5h/11m		They can cleave GSDMD into N-GSDMD, but can not cleave pro-IL-1β/pro-IL-18. Nevertheless, they can facilitate the maturation and secretion of IL-1β/IL-18 through the NLRP3/caspase-1 pathway.
		caspase-12		undefined.
		caspase-13(bovine)		unknown
Apoptotic Caspase	initiator caspase	caspase-2	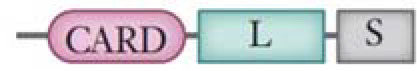	Its function is described to be cell-cycle related.
		caspase-8\10	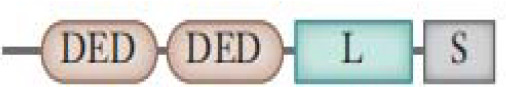	To initiate apoptosis via activating the executioner caspases-3, -6, and -7.
		caspase-9	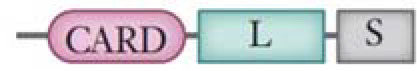	
	effector caspases	caspase-3	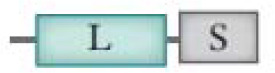	They are responsible for the characteristic morphological changes of apoptosis.
		caspase-6\7		
undefined		caspase-14	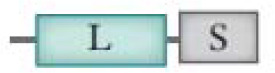	It is linked to cell differentiation.

m, murine and h, human.

Apoptotic caspases are instrumental in initiating and executing apoptosis, a tightly regulated process that typically does not trigger an immune response (164). These caspases can be further divided into initiator and effector caspases based on their roles in the apoptotic pathway (164–166). Initiator caspases, such as caspase-2, -8, -9, and -10, act as proteolytic signal amplifiers, responsible for activating the downstream effector caspases. Effector caspases, including caspase-3, -6, and -7, are tasked with cleaving a variety of cellular proteins at specific sites, thereby facilitating the progression of apoptosis.

In contrast, inflammatory caspases, which include caspase-1, -4, -5, and-11. Research has indicated that Caspase-1 plays a critical role in orchestrating the canonical pyroptotic cascade, while Caspase-4 and -5 in humans, along with Caspase-11 in murine models, govern the noncanonical pyroptotic pathway (163, 165). The roles of caspase-12, -13, and -14 are less defined and are subjects of ongoing research (161, 163). Interestingly, Caspase-3 is primarily associated with apoptosis, but recent studies have suggested that influenced by TNF or various chemotherapeutic agents, GSDME may induce a transition from apoptotic to pyroptotic cellular death through Caspase-3 modulation (29).

### Caspase-1 in IIM

In IIM, the caspase-1-mediated canonical pathway has been identified as a crucial element in the pathogenesis. Caspase-1 is a key enzyme responsible for protein hydrolysis in the pyroptosis pathway. In the cytoplasm, pro-caspase-1 exists initially in an inactive state. Upon stimulation by various factors, it is activated from its inactive pro-form pro-caspase-1 to an active state by the inflammasome complex, leading to its self-cleavage into p20 and p10 subunits, forming an active p20/p10 heterodimeric enzyme. Activated caspase-1 converts pro-interleukins, such as pro-IL-1β and pro-IL-18, into their active forms IL-1β and IL-18, enhancing the inflammatory response, cleaving GSDM D into N-terminal and C-terminal fragments, thus leading to pyroptosis (166). This activation is crucial for regulating cell death and inflammatory responses, emphasizing its role in IIM (167).

In DM/PM patients, studies have shown that caspase-1 is mainly located in the muscle fiber sarcolemma and is associated with sites of tissue regeneration (167). The distribution of active caspase-1 is similar to IL-1β, with increased levels of caspase-1 mRNA and caspase-1 p20 protein (64, 82). Experimental autoimmune myositis (EAM) mouse models also show a significant increase in the expression of caspase-1 mRNA and proteins, including pro-caspase-1 and its cleaved form caspase-1 p20, compared to control groups (64). These findings suggest that caspase-1 is involved in the activation of proIL-1β and plays a role in cell proliferation.

Death cells may serve as a repository of self-antigens, triggering systemic autoimmunity in susceptible individuals. Anti-PM/Scl autoantibodies are common in patients with overlap myositis and scleroderma, targeting the exosome subunit PM/Scl-75. Schilders demonstrated that cleavage of PM/Scl-75 was inhibited by caspase inhibitors, with caspase-1 being the most effective, followed by caspase-8, while inhibition by caspase-3 and -7 was weaker (168). Cleavage occurs at Asp369 at the C-terminal, with the N-terminal fragment still attached to the exosome (168). Follicular T helper (TFH) cells participate in B cell differentiation and autoantibody production. In anti-MDA5 positive IIM patients, there is a significant increase in active caspase-1 on TFH cells, suggesting a role for caspase-1 on TFH cells in the pathogenesis of anti-MDA5 positive IIM and potentially serving as a disease biomarker for this patient subgroup (169).

### Caspase-1 inhibitors

Comprehensive research has highlighted the key role of caspase-1 in the pathophysiology of IIM (64, 66, 82, 108, 141, 167–169), indicating its potential as a biomarker of disease activity and therapeutic target (**Table 5**). The discovery of small PYD or CARD-only proteins (POPs and COPs) has introduced new regulatory mechanisms. Devi S et al. demonstrated that COPs can regulate the activation of inflammasomes by altering CARD-CARD interactions, thereby reducing the activation of NLRP3 inflammasomes and disease progression (173). Zhou Y et al. showed the therapeutic potential of extracellular vesicles containing a caspase-1 inhibitor (EVs-VX-765) derived from dendritic cells, which significantly suppressed levels of interleukin-1β and inhibited Th17 responses and follicular reactions. This approach holds promise in the treatment of myasthenia gravis and other autoimmune diseases, including IIM (174). CZL80 is a compound with a strong affinity for the active site of caspase-1 (175). AC-YVAD-CMK is a selective caspase-1 inhibitor that reduces the expression of NLRP1 inflammasomes and alleviates the release of IL-1β and IL-18 induced by NLRP1 inflammasomes (176) (**Table 6**).

**Table 5:** Structure, target, and mechanism of potential inhibitors of caspase-1.

Agent	Structure	Target	Potential mechanism	References
VX-765	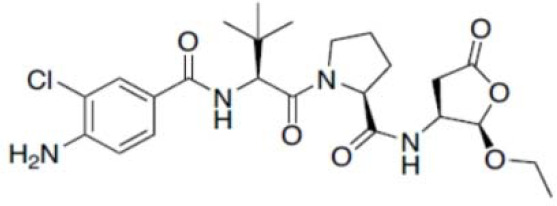	Caspase-1	Suppresses interleukin-1β levels and inhibits Th17 responses and germinal center reactions.	(170)
CZL80	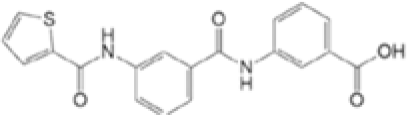	Caspase-1	It is a structure‐matching compound with a strong affinity to the active sites of caspase‐1.	(171)
AC-YVAD-CMK	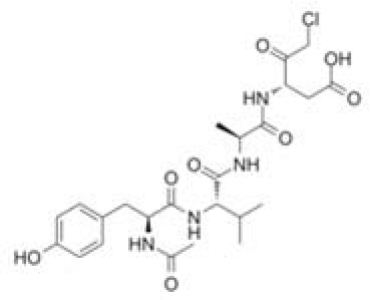	selective caspase-1 inhibitor	decrease the expression of NLRP1 inflammasome, and relieve the release of IL-1β and IL-18 induced by NLRP1 inflammasome.	(172)

**Table 6:** Characteristics of gasdermin family members (177–180).

Protein	Chromosome	Normal human tissue expression	Activation mechanism	Biological function
sGSDMA	17q21.1 (H), 11D (M)	Gut epithelium, skin, mammary gland, and kidney	Streptococcus pyogenes (SpeB virulence factor)	It is associated with mitochondrial homeostasis. Gasdermin A gene mutation can cause alopecia, asthma, local cutaneous sclerosis, and inflammatory bowel disease.
GSDMB	17q21 (H)	Esophagus and gastrointestinal epithelium, respiratory system, liver, colon, and lymphocytes	Caspase 1; granzyme A; caspase-3, -6 and -7 *in vitro*	It is associated with pyroptosis and anti-tumor immunity. Gasdermin B mutation can cause breast cancer and asthma
GSDMC	8q24.21 (H), 15D1 (M)	Stomach, small intestine, cecum and colon epithelium, airway epithelium, skin, spleen, vagina, and bladder	Caspase 8	GSDMC/caspase-8 mediates a non-canonical pyroptosis pathway in cancer cells, causing tumor necrosis.
GSDMD	8q24.3 (H), 15D3 (M)	Immune cells, liver, gut epithelium, kidney	Caspases 1, 4 and 5 (h); caspases 1 and 11 (m); caspase 8; cathepsin G; neutrophil elastase	It is associated with pyroptosis which is crucial for maintaining immune homeostasis, host defense against infections and various inflammation, such T2DM, cryopyrin-associated periodic syndromes, sepsis and autoinflammatory (25, 41–43).
GSDME (DFNA5)	7p15.3 (H), 6B2.3 (M)	Placenta, heart, brain and kidney	Caspases 3, 7, and 8; granzyme B	It is associated with hearing impairment, cancer, and autoimmune disease.
PJVK (DFNB59)	2q31.2 (H), 2C3 (M)	Auditory system, including neurons, hair cells, supporting cells, and spiral ganglion cells in the inner ear	Unknown	It is associated with hearing impairment in humans and mice.

m, murine and h, human.

### Human caspase-4, -5, or mouse caspase-11 in IIM

Human caspase-4 and -5, as well as their murine homolog caspase-11, are critical cysteine proteases in immune responses, particularly in inflammasome complexes activation (170–172, 181). These enzymes are crucial for sensing cytoplasmic lipopolysaccharide (LPS) from Gram-negative bacteria, which is a major trigger of inflammation.

Caspase-4/5/11 is activated by binding to LPS through its caspase recruitment domain (CARD), leading to its aggregation and self-cleavage, forming noncanonical inflammasomes. This process operates without additional adapter proteins or cofactors, making caspase-4/5/11 sensors and effectors in immune responses (23). Once activated, these caspases initiate proteolytic processing of the ubiquitin-1 transmembrane channel, promoting ATP release and subsequent activation of the P2X7 receptor channel. They also cleave GSDMD to form membrane pores, triggering pyroptosis, a programmed cell death that facilitates the release of inflammatory cytokines such as interleukin-1β (IL-1β) and IL-18, crucial for defending against bacterial infections. Notably, caspase-4/5/11 does not process pro-IL-1β into its mature form (22, 182); instead, it induces cell death through GSDMD cleavage, and the release of IL-1β depends on subsequent caspase-1 activation (183, 184).

Elevated caspase-4, caspase-5, GSDMD, NLRP3, ubiquitin-1, and purinergic receptor P2X7 were observed in the muscles of DM and PM patients. The positive correlation between muscular tissue pathology scores and these protein markers suggests a direct link between muscle pathology and their expression (185).

In mice, the activation of caspase-11 is driven by type I interferons (IFNs), and its activation is weakened in the absence of type I IFN signaling during bacterial infection (186–188). This response is crucial for caspase-11 to interact with its ligands, as IFN-induced GTPases disrupt pathogen-containing vacuoles, allowing caspase-11 to detect cytosolic LPS (120). Human guanylate-binding protein-1 (hGBP1) assists caspase-4 in detecting LPS through a “detergent-like” mechanism, exposing lipid A in the outer membrane of Gram-negative bacteria (189–191). Another pathway for cytosolic LPS entry involves host proteins, with HMGB1-bound LPS mediating caspase-11 activation (189). Caspase-11 promotes potassium efflux through GSDMD cleavage and pore formation, leading to the activation of NLRP3 and the release of IL-1β.

Apart from detecting LPS, noncanonical caspases also possess pattern recognition receptor (PRR) activity; for instance, caspase-11 can bind oxidized phospholipids (oxPAPC) derived from dying cells as damage-associated molecular patterns (DAMPs) to trigger IL-1β release and pyroptosis (192). However, other studies have shown that oxPAPC binds caspase-4 and caspase-11 to inhibit the activation of noncanonical inflammasomes (193). These conflicting results may stem from differences in specific oxPAPC moieties, which require further investigation (194).

Understanding the roles of caspase-4, caspase-5, and caspase-11 in myositis and other inflammatory conditions is crucial for the development of targeted therapies. Modulating their activities may help reduce inflammation and muscle damage, or enhance pathogen clearance, providing potential therapeutic options for inflammatory muscle diseases.

## Gasdermins

Gasdermin (GSDM) is the executor of pyroptosis. GSDM was initially discovered in the gastrointestinal tract and skin, hence the name (“gas-dermin”). GSDMs are a functionally diverse family of proteins, including GSDMA, GSDMB, GSDMC, GSDMD, GSDME (or DFNA5), and PJVK (DFNB59), which are expressed in multiple cell types and tissues (195) (**Table 5**). GSDM is the core of pyroptotic execution, mediating the formation of membrane pores and leading to cell lysis (196). Except for PJVK (DFNB59), whose function remains unclear, these proteins all contain an N-terminal domain with the intrinsic ability to form necrotic pores and a C-terminal domain that regulates cell death inhibition through interactions within the molecule (23, 177, 197). Protein proteolytic cleavage occurs at the junction between the N-terminal and C-terminal domains of GSDM, releasing the C-terminal domain, thereby promoting translocation of the N-terminal domain of GSDM to the cell membrane, where it aggregates to form pores (23, 177, 197). The N-terminal fragments of GSDMs have a high affinity for phosphatidylinositol phosphates located in the inner leaflet of the plasma membrane and a lower affinity for phosphatidylserine (178). Additionally, GSDMs can also target cardiolipin primarily found in mitochondrial and bacterial membranes (196).

### GSDMD and GSDME in IIM

GSDMD is the most extensively studied member of the GSDM family, located in the 8q24 region of chromosome 8. This protein is widely distributed in various tissues and immune cells. Both human and mouse GSDMD consist of a 31kDa NT pore-forming domain and a 22kDa CT inhibitory domain, connected by a domain containing a cleavage site. This connector is cleaved by Canonicalal caspase-1 or non-Canonicalal caspase-4/5/11 upon activation of various inflammasomes, leading to the separation of GSDMD-NT and GSDMD-CT. GSDMD-NT is inserted into membranes and polymerizes to form pores, releasing cytokines (179, 180, 198). GSDMD, as a key executor of pyroptosis, plays a crucial role in the development and progression of various inflammatory diseases, autoimmune diseases, and many other systemic diseases (199).

Recent studies have found that the expression levels of Caspase-4, Caspase-5, Caspase-11, and GSDMD proteins in the skeletal muscle tissue of the EAM group were higher than those in the control group. Additionally, the mRNA expression levels of Caspase-11 and GSDMD in the EAM group were higher than those in the control group. Furthermore, the levels of serum IL-18 and IL-1β detected in mice in the EAM group were higher than those in the control group, suggesting that the non-Canonicalal mediated activation of the GSDMD inflammatory necrosis pathway may be related to the pathogenesis of IIM (67).

Interstitial lung involvement is a prominent feature of IIM-ILD, and endothelial injury may play a key role in the leakage. In the experimental autoimmune myositis (EAM) model in mice, the expression of downstream joint protein ASC, activated Caspase-1 fragment, pyroptosis protein GSDMD, and the related inflammation factor IL-1β significantly increased in pulmonary microvascular endothelial cells (PMECs), and this was also observed after *in vitro* stimulation with NETs, suggesting that NETs-induced pyroptosis occurs through the canonical pyroptosis pathway (110).

GSDME, historically known as DFNA5, is associated with hereditary hearing loss (198). It is activated by cleavage of Caspase-3, promoting the transition from apoptotic pathway to pyroptosis. The activation of GSDME is related to the mitochondrial apoptotic pathway, and when cells with high GSDME expression undergo chemotherapy, apoptosis may transition to pyroptosis (29, 200). Liu et al. found GSDME and Caspase-3 in the muscle tissue immunohistochemical staining of DM patients, providing preliminary evidence for the association between GSDME-dependent pyroptosis and muscle bundle atrophy (201).

### GSDMD inhibitors

Given that GSDMD is a crucial protein in executing pyroptosis and is associated with inflammation signaling, activation of various inflammatory bodies, and the release of downstream inflammatory cytokines, inhibiting its activation is thought to be an effective approach to managing related inflammatory conditions. In recent years, several small synthetic molecular inhibitors have been found to inhibit GSDMD-mediated pyroptosis through different mechanisms (202, 203) (**Table 7**).

**Table 7:** Structure and mechanism of potential inhibitors of GSDMD inflammasome.

Agent	Structure	Potential mechanism	References
Disulfiram (DSF)	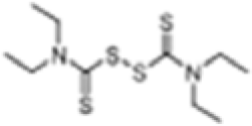	Modifying Cys191 of GSDMD and inhibiting the oligomerization of GSDMD-NT	(204)
Necrosulfonamide (NSA)	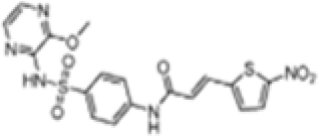	Binding directly to Cys191 of GSDMD and inhibiting the oligomerization of GSDMD-NT	(205)
Dimethyl fumarate (DMF)	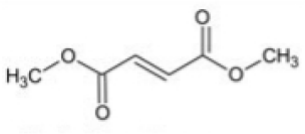	Succinating Cys191 of GSDMD,blocking caspase-GSDMD interactions and inhibiting the oligomerization of GSDMD-NT	(206)
C202-2729	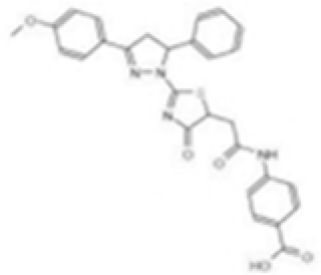	Binding directly to GSDMD-NT and inhibiting the oligomerization of GSDMD-NT	(207)
GI-Y1	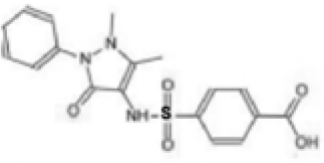	Target Arg7 residue of GSDMD-N, inhibit GSDMD-mediated lipid-binding, pore formation and mitochondrial binding	(208)
LDC7559	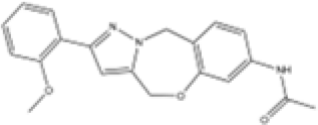	Inhibit the cleavage of GSDMD in neutrophil cell death pathway (NETosis)	(209)

Disulfiram(DSF), a drug for treating alcohol addiction, covalently modifies human/mouse Cys191/Cys192 in GSDMD and inhibits the oligomerization of GSDMD-NT (204). Necrosulfonamide (NSA) is a small molecule that directly binds to Cys191 of GSDMD, preventing the aggregation of GSDMD-NT, especially blocking MLKL, to inhibit plasma membrane rupture and reduce cell death (205). Dimethyl fumarate (DMF) is known for its anti-inflammatory and immune-modulating properties, and studies have found its effectiveness in treating diseases such as multiple sclerosis (MS) and psoriasis. Although the exact mechanism is unclear, research has shown that DMF modifies Cys191 of GSDMD and inhibits the polymerization of GSDMD-NT (206). Furthermore, DMF is marketed under the brand name Tecfidera for psoriasis management. NSA exhibits significant therapeutic benefits in various diseases. C202-2729 is a small molecule known for its strong anti-inflammatory effects. Its efficacy has been demonstrated in mouse models of endotoxin shock and experimental autoimmune encephalomyelitis (EAE), indicating its potential in treating inflammatory diseases (207). The mechanism of action of C202-2729 involves the direct binding of the N-terminus of GSDMD to inhibit GSDMD activation, thereby preventing the translocation of the N-terminal GSDMD fragment to the cell membrane, inhibiting pore formation, and the release of mature IL-1β, which is a key pro-inflammatory cytokine (207). In 2023, a new GSDMD inhibitor, GI-Y1, was discovered through virtual and pharmacological screening. GI-Y1 inhibits GSDMD-mediated lipid binding, pore formation, and mitochondrial binding by targeting the Arg7 residue of GSDMD-N in cardiac muscle cells, suggesting its potential cardioprotective effects against myocardial ischemia/reperfusion injury (208). LDC7559 is an inhibitor of neutrophil extracellular traps, which binds to GSDMD and inhibits NETosis. Mechanistically, LDC7559 can neutralize the toxicity of GSDMD-N in humans and mice, showing a direct inhibitory effect on GSDMD activity (209).

In conclusion, these findings underscore the key roles of GSDMD and GSDME in the pathogenesis of pyroptosis, closely associated with the occurrence and progression of DM and PM. Inhibitors of GSDMD may present novel therapeutic opportunities for the management of IIM. The potential to modulate GSDM proteins offers a hopeful avenue for the development of treatment strategies aimed at alleviating the burden of IIM by interrupting the pyroptosis cascade reaction.

## Conclusion and prospects

The pathogenesis of IIM is complex, involving the interplay of immune dysregulation, inflammation, and cellular stress. Pyroptosis has emerged as a key pathological process in IIM, with NLRP3 and AIM2 inflammasomes, caspases, and gasdermin proteins playing central roles, which may offer new therapeutic strategies for IIM. Future directions in IIM research should include detailed explorations into the molecular mechanisms underlying pyroptosis, the development of targeted therapies towards the pyroptotic pathway, and evaluation of the efficacy and safety of these interventions in clinical trials. Understanding the interactions between inflammasome-mediated pyroptosis and other pathways, such as NETs, autophagy, and endoplasmic reticulum stress, may reveal additional therapeutic targets. The potential of immune metabolic pathways in regulating pyroptosis also warrants exploration. Ultimately, a deeper understanding of inflammasomes in IIM may lead to more effective treatment approaches and improved patient outcomes.
